# Genome-wide identification, bioinformatics and expression analysis of HD-Zip gene family in peach

**DOI:** 10.1186/s12870-023-04061-w

**Published:** 2023-03-02

**Authors:** Zhe Wang, Xuelian Wu, Binbin Zhang, Yuansong Xiao, Jian Guo, Jin Liu, Qiuju Chen, Futian Peng

**Affiliations:** 1 State Key Laboratory of Crop Biology, College of Horticulture Science and Engineering, Shandong Agricultural University, 271000 Tai’an, China; 2 Agricultural Technical Service Center of Yiyuan County, 256100 Zibo, China

**Keywords:** Peach, HD-Zip transcription factor, Root formation, Genome-wide analysis

## Abstract

**Background:**

HD-Zips (Homeodomain-Leucine Zippers) are a class of plant-specific transcription factors that play multiple roles in plant growth and development. Although some functions of HD-Zip transcription factor have been reported in several plants, it has not been comprehensively studied in peach, especially during adventitious root formation of peach cuttings.

**Results:**

In this study, 23 *HD-Zip* genes distributed on 6 chromosomes were identified from the peach (*Prunus persica*) genome, and named *PpHDZ01-23* according to their positions on the chromosomes. These 23 PpHDZ transcription factors all contained a homeomorphism box domain and a leucine zipper domain, were divided into 4 subfamilies(I-IV) according to the evolutionary analysis, and their promoters contained many different cis-acting elements. Spatio-temporal expression pattern showed that these genes were expressed in many tissues with different levels, and they had distinct expression pattern during adventitious root formation and development.

**Conclusion:**

Our results showed the roles of PpHDZs on root formation, which is helpful to better understand the classification and function of peach *HD-Zip* genes.

**Supplementary Information:**

The online version contains supplementary material available at 10.1186/s12870-023-04061-w.

## Introduction

HD-Zip transcription factors belong to the Homeodomain (HD) transcription factor superfamily and are only found in plants. HD-Zip protein usually contain two highly conserved domains: homeodomain (HD) and leucine zipper (LZ) [1]. The homeodomain domain is generally composed of about 60 amino acids encoded homeobox, which form three alpha helices. The three helices formed a tight spherical structure with hydrophobic core under the action of two loops and one corner [2]. And the arrangement of the three helices determines the difference in the specific binding of homeodomain to DNA. The leucine zipper domain forms a dimer that is essential for HD-Zip protein recognition of DNA [3]. According to the differences in structure and function, HD-Zip family members can be divided into four subfamilies: I, II, III and IV. The four subfamilies all have HD and LZ domains. In addition to HD and LZ domains, subfamily IV also has a START domain related to sterol binding. Compared with subfamily IV, subfamily III has another C-terminal MEKHLX domain [4].

HD-Zip transcription factors have been identified in a variety of plants, such as *Arabidopsis thaliana* [5], *Zea mays* [6] *Oryza sativa* [7], *Populus trichocarpa* [8], and so on. As a unique transcription factor in plants, HD-Zip plays an important regulatory role in plant growth and development, environmental response, cell cycle and cell metabolism [9]. For example, NaHD20 could regulate ABA accumulation and activate the expression of dehydration related genes in tobacco leaves under water stress [10]. In *Arabidopsis thaliana*, overexpression of *ATHB17* can increase chlorophyll content and significantly improve photosynthetic capacity [11]. *PtrHB7* overexpression enhanced the differentiation of cambium cells into xylem cells and balanced the differentiation of secondary xylem and phloem [12]. ZmOCL1 may regulate the development of corn kernels by regulating gibberellin level [13].

As one of the most important fruits in the world with high nutritional value, peach is widely grown all over the world. In our country, seedling rootstock grafting is the main method of peach propagation. However, due to the obvious separation of traits in the offspring and the poor uniformity of seedlings, it brings many problems to production management. Cutting propagation can preserve the excellent characters of mother plant, and can produce a large number of seedlings in a short period of time, so the cutting propagation of peach has a good application prospect. The formation and growth of adventitious root is the key to cutting propagation, which is regulated by genes. Many studies have found that HD-Zip proteins are involved in the formation and development of roots. Such as MtHB1 that regulate the emergence of lateral roots by mediating *LBD1* expression in alfalfa [14], HAT2 that negatively regulates root growth, reducing lateral root length and ATHB10 regulating root hair development by participating in ethylene dependent pathway in *Arabidopsis thaliana* [15, 16]. The HD-Zip III subfamily in *Arabidopsis thaliana* regulates root development through plant hormones [17]. However, the roles of HD-Zips in root formation and development of peach has not yet been studied.

In this study, the HD-Zip transcription factor family of peach was analyzed. A total of 23 *HD-Zip* genes were identified in peach genome, and their chromosome localization, phylogenetic relationship, gene structure, conserved domain and promoter elements were analyzed. Meanwhile, we also investigated the expression patterns of *PpHDZ* genes in different tissues and in the process of adventitious root development. These results provided a basis for further study on the function of PpHDZ family members in peach.

## Results

### Identification of HD-Zip gene family members in peach

**Fig. 1:**
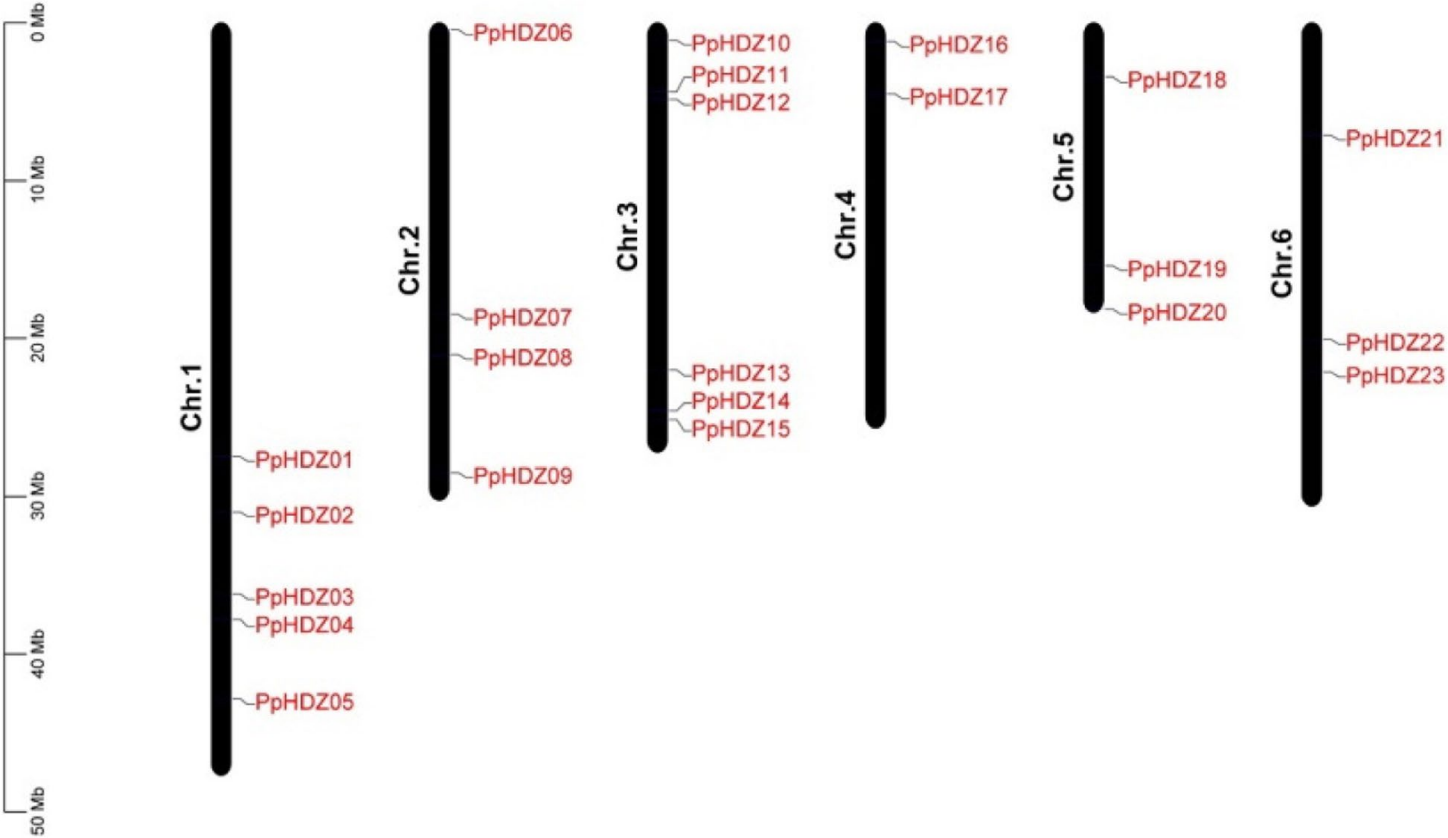
The location of* HD-Zip* gene on the peach chromosome**. **Chromosomes 1-6 are shown in long black bars. The approximate distribution of each *PpHDZ* gene is marked in red font on a long black bar.

The *HD-Zip* gene in peach was identified by whole-genome analysis method, and the HMM model was constructed according to the characteristic domain of HD-Zip protein (PF00046). The HMM model was used to search peach genome to obtain candidate genes, and NCBI was used to further detect whether their sequences contained the HD-Zip domain. Finally, 23 *HD-Zip* genes were identified, named *PpHDZ01-23* according to their location on the chromosomes. These 23 *PpHDZ* genes were unevenly distributed on 6 chromosomes (Fig. 1). The number of *PpHDZ* genes on Chr. 3 was up to 6, followed by 5, 4, 3 and 3 *PpHDZ* genes on Chr. 1, Chr. 2, Chr.5 and Chr. 6, respectively, and there were at least 2 *PpHDZ* genes on Chr. 4.

The predicted proteins of *PpHDZ* genes contain amino acids ranged from 227 (PpHDZ02) to 849 (PpHDZ17), with an average amino acid number of 556. The molecular weight ranged from 25,495.91 Da (PpHDZ02) to 93,018.86 Da (PpHDZ17), and the average molecular weight was 61,445.54 Da. The isoelectric point (pI) is an important physiological indicator of a protein, which mainly depends on the ratio of the number of acidic amino acid to basic amino acid. The theoretical isoelectric point of PpHDZs were between 5.4182 and 9.2656 (Table 1). The isoelectric point of most PpHDZ proteins (87%) was less than 7, which indicates that PpHDZ proteins might be acidic proteins.

**Table 1:** Physical and chemical properties of peach HD-Zip gene family members

Gene name	Gene accession	aa	Chrom	Chr-srart	Chr-end	MW(Da)	pI	E-value
PpHDZ01	Prupe.1g267300	321	Chr.1	27,479,047	27,481,525	36,193.92	4.4513	1.2E-17
PpHDZ02	Prupe.1g325000	227	Chr.1	30,996,031	30,998,955	25,495.91	9.2656	2.79E-16
PpHDZ03	Prupe.1g416800	311	Chr.1	36,184,611	36,186,254	35,033.98	6.5401	1.17E-15
PpHDZ04	Prupe.1g447100	840	Chr.1	37,799,927	37,806,131	92,410.75	6.2714	4.39E-17
PpHDZ05	Prupe.1g523000	293	Chr.1	42,838,766	42,840,575	33,288.79	5.7841	9.51E-16
PpHDZ06	Prupe.2g004000	368	Chr.2	413,733	415,799	40,137.10	6.7606	1.59E-16
PpHDZ07	Prupe.2g127300	300	Chr.2	18,480,783	18,483,560	33,391.38	7.5154	3E-14
PpHDZ08	Prupe.2g156900	829	Chr.2	21,026,929	21,033,200	90,225.21	6.3463	1.72E-18
PpHDZ09	Prupe.2g291400	745	Chr.2	28,494,027	28,500,411	81,863.13	6.1757	1.18E-19
PpHDZ10	Prupe.3g015300	685	Chr.3	1,087,426	1,091,967	76,060.19	6.4549	1.39E-17
PpHDZ11	Prupe.3g060700	837	Chr.3	4,364,306	4,371,314	91,800.46	6.4620	7.27E-17
PpHDZ12	Prupe.3g067200	757	Chr.3	4,843,493	4,848,265	84,381.51	6.0886	5.46E-17
PpHDZ13	Prupe.3g218500	756	Chr.3	21,997,930	22,003,936	82,420.17	5.7489	2.17E-19
PpHDZ14	Prupe.3g283200	279	Chr.3	24,595,564	24,602,251	31,813.72	4.5535	2.85E-19
PpHDZ15	Prupe.3g269900	306	Chr.3	25,144,835	25,147,011	34,762.64	6.9138	2.19E-16
PpHDZ16	Prupe.4g024800	750	Chr.4	1,177,456	1,183,177	82,035.70	6.2360	4.93E-19
PpHDZ17	Prupe.4g090100	849	Chr.4	4,481,030	4,488,275	93,018.86	6.2426	2.35E-16
PpHDZ18	Prupe.5g029000	831	Chr.5	3,415,709	3,421,770	92,217.28	5.4147	4.56E-20
PpHDZ19	Prupe.5g185900	329	Chr.5	15,407,945	15,410,256	36,763.21	4.4182	1.09E-17
PpHDZ20	Prupe.5g240900	709	Chr.5	18,150,720	18,154,548	78,187.84	6.6245	3.33E-18
PpHDZ21	Prupe.6g102300	842	Chr.6	7,124,615	7,132,194	92,159.97	6.4276	1.27E-16
PpHDZ22	Prupe.6g193400	337	Chr.6	20,066,352	20,068,990	38,048.23	4.9816	1.24E-15
PpHDZ23	Prupe.6g213800	285	Chr.6	22,151,333	22,152,861	31,537.37	8.3918	6.43E-17

*Aa* amino acid, Chrom chromosome location, *MW* molecular weight, *pI* theoretical isoelectric point

### Phylogenetic relationships of peach HD-Zip gene family

**Fig. 2:**
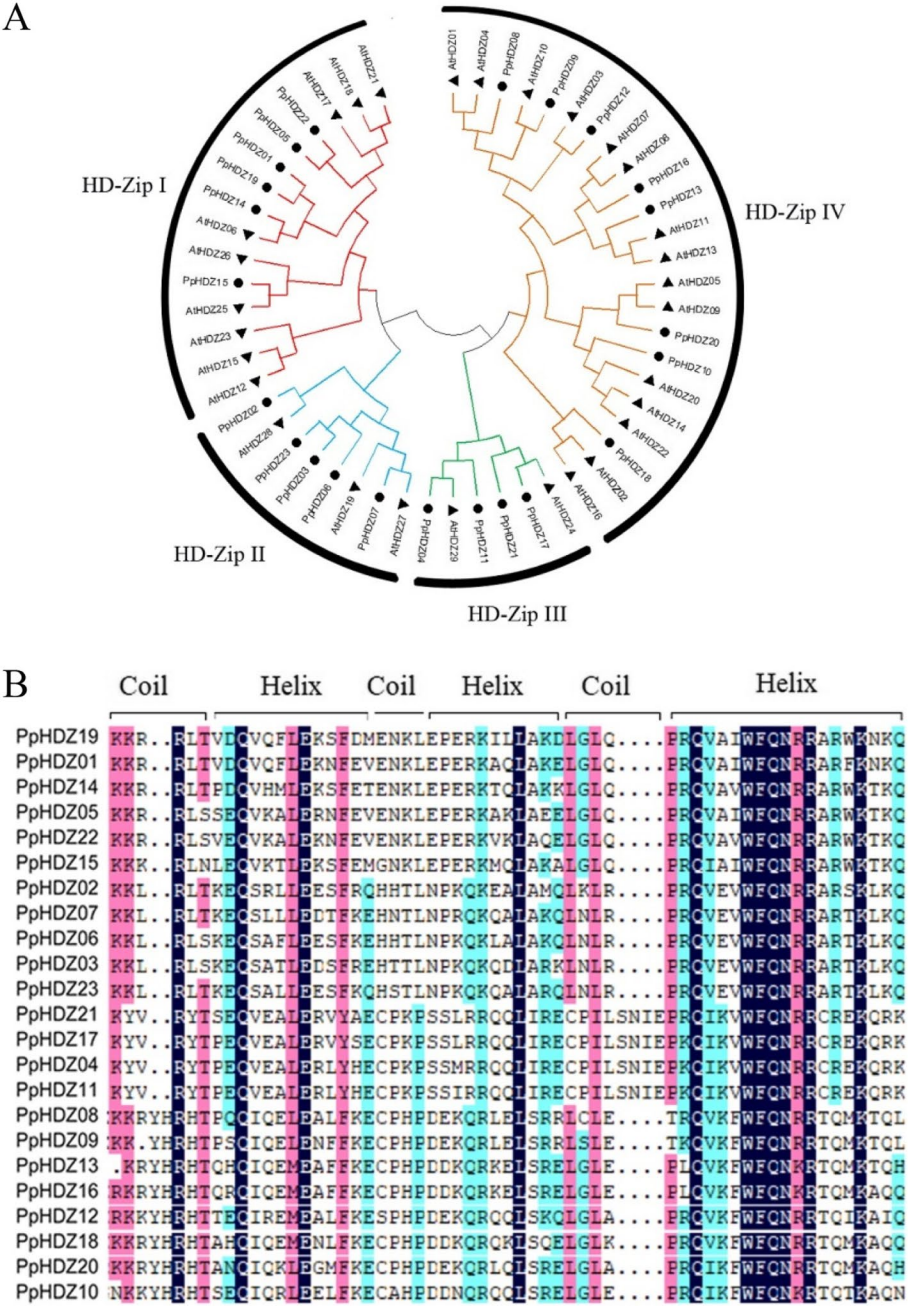
Phylogenetic analysis of HD-Zip family among peach and Arabidopsis.** A** Phylogenetic tree of peach and Arabidopsis HD-Zip family. The triangle represents *Arabidopsis thaliana* and the circle represents the peach. **B** Multiple sequence alignment of peach HD-Zip protein

In order to understand the homology of 23 *PpHDZ* genes in peach and predict their functions, phylogenetic tree was constructed with Arabidopsis HD-Zip gene families. It showed that peach HD-Zip gene family could be divided into four subfamilies (I-IV), which included 6, 5, 4, 8 HD-Zip gene family members respectively, and subfamilies I and II were closely related and in the same branch, (Fig. 2 A). All the PpHDZ proteins contained a HD domain, which were composed of 3 coil-helix structures (Fig. 2B). And the amino acid sequence alignment of HD domains showed that the amino acid number of subfamilies I and II in their characteristic structural domain is the same, with 4 amino acids less than that of subfamily III and 2 amino acids less than that of subfamily IV.

Through phylogenetic tree and amino acid sequence alignment in HD domain, we could infer the function of *PpHDZ* genes of peach from the function of *HD-Zip* genes in *Arabidopsis thaliana*, a model plant with known function.

### Analysis of conserved motifs, domains and number of exons

**Fig. 3:**
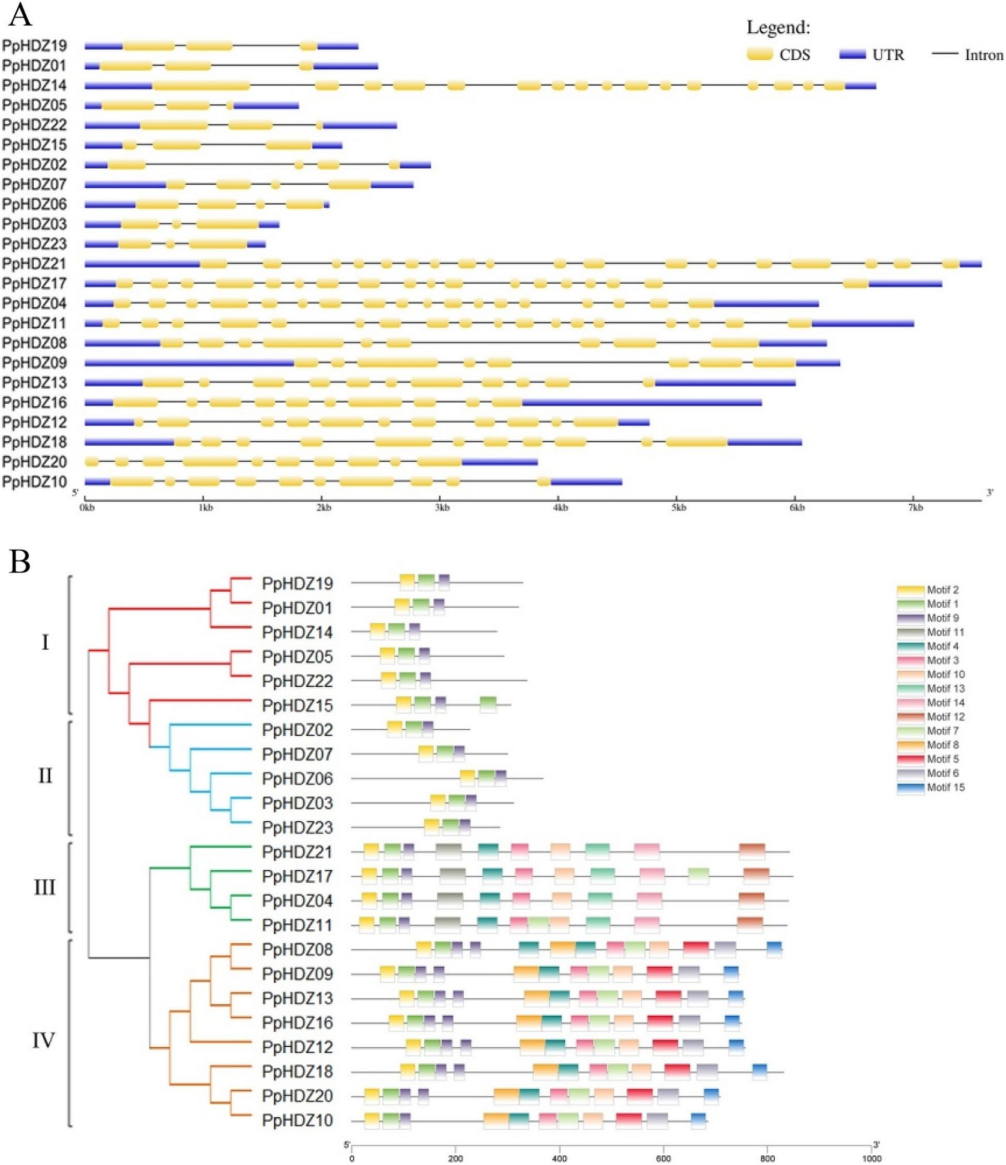
Phylogeny, gene structure and conserved motifs analysis of peach HD-Zip family.** A** Exon-intron structure of *PpHDZ* gene. **B** PpHDZ family gene phylogenetic tree and protein conserved motifs distribution

In order to further understand the evolutionary relationship of peach HD-Zip protein, the structure of peach HD-Zip gene family was analyzed by GSDS. There were significant differences in gene structure among the four subfamilies of peach HD-Zip gene family, and the number of exon and intron in the same subfamily was similar. The gene coding sequence of subfamilies I and II was simpler than that of subfamilies III and IV. The number of exon in subfamilies I and II members was 3–4, while that of subfamilies III and IV members was more than 10 (Fig. 3 A). These findings suggested that the exons of the HD-Zip gene family were lost or increased during evolution.

TBtools was used to analyze the protein conserved domain. It was showed that *HD-Zip* genes from the same subfamily had the same domain distribution and composition, suggesting that members of the same subfamily might have similar functions. All members of peach HD-Zip gene family had HD and LZ domains, that is, Motif 1, 2, 9 together constitute the HD-Zip domain, suggesting that these two domains play an important role in the expression of *HD-Zip* gene. There were only HD and LZ domains in subfamilies I and II. Both subfamilies III and IV had a START domain related to sterol binding, named Motif 3, 4, 10, 11. Subfamily III also had a special C-terminal MEKHLA domain, named Motif 12 (Fig. 3B). This is consistent with previous reports that the existence of these subfamily-specific domains may be related to subfamily-specific functions [18–20]. Therefore, the analysis of conserved motif and exon composition further supported the study of the evolution of HD-Zip gene family.

### Gene replication and collinearity analysis of *PpHDZ* gene

**Fig. 4:**
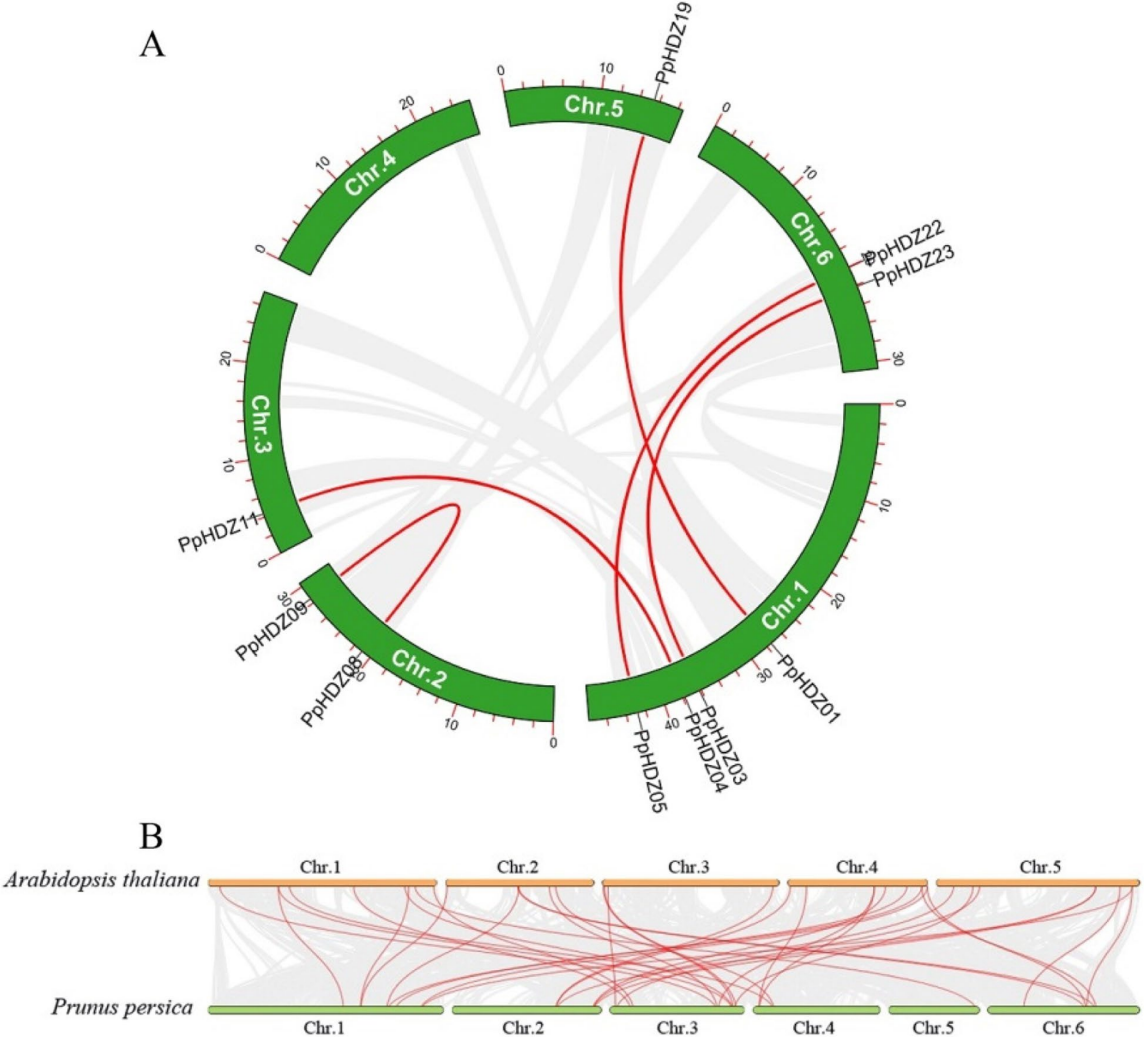
Collinearity analysis.** A** Collinearity analysis of *HD-Zip* gene in peach species. **B** *HD-Zip* gene collinearity between peach and Arabidopsis genomes

In order to investigate the expansion and evolution mechanism of PpHDZ gene family, gene replication events in peach genome were studied. A total of 5 pairs of homologous genes were found in peach chromosomes, which were *PpHDZ01/PpHDZ19, PpHDZ03/PpHDZ23, PpHDZ04/PpHDZ11, PpHDZ05/PpHDZ22* and *PpHDZ08/PpHDZ09* (Fig. 4 A). This suggested that segmental duplication was the main cause of PpHDZ gene family amplification. In addition, to further understand the origin and function of the PpHDZ gene family, we also mapped the collinearity of *HD-Zip* gene in the peach and Arabidopsis genomes. A total of 37 pairs of *HD-Zip* genes were identified in peach and Arabidopsis (Fig. 4B). This showed that *HD-Zip* gene in peach and Arabidopsis has high homology, indicating that *HD-Zip* gene had high conservation.

### GO annotation analysis

**Fig. 5:**
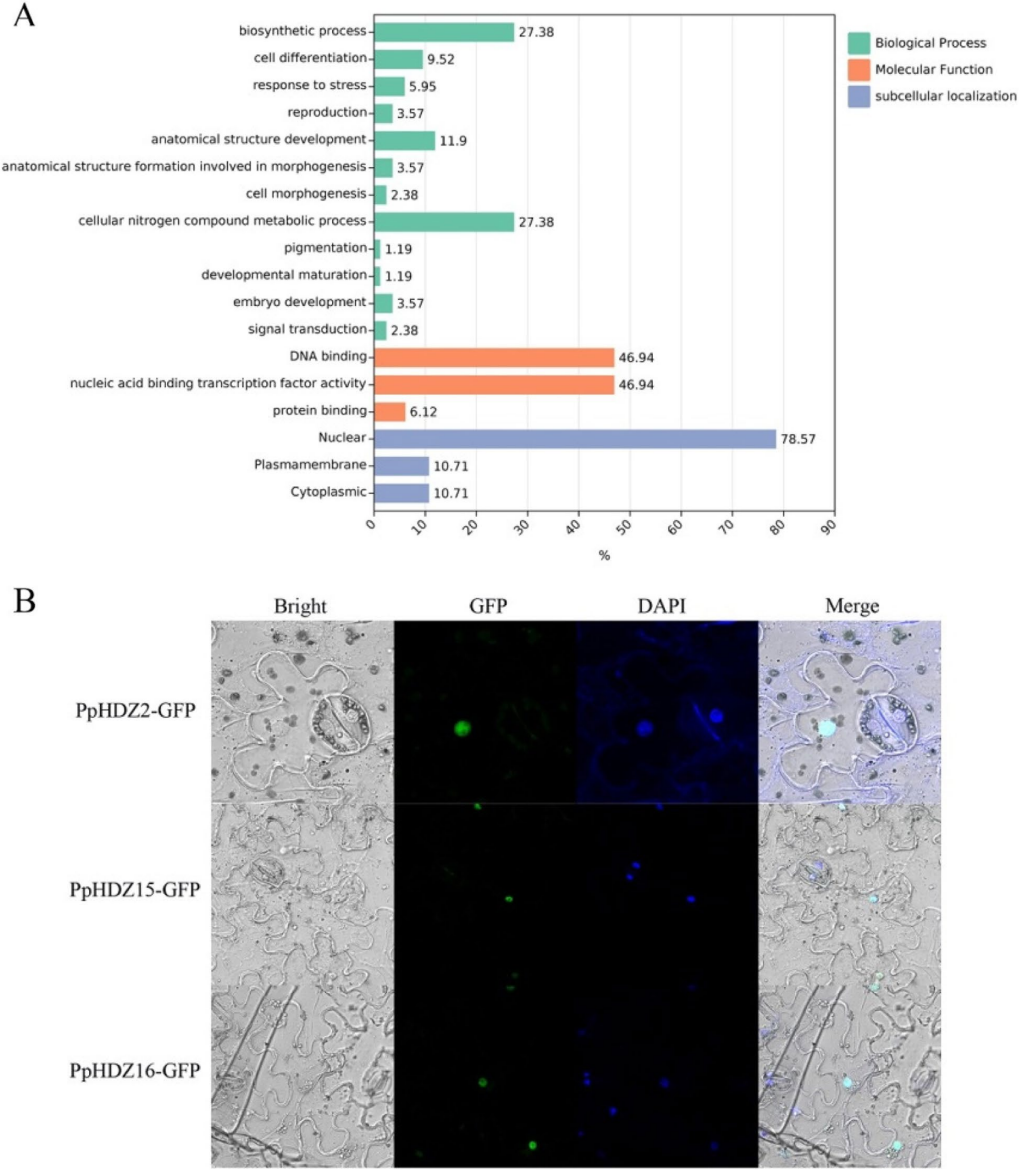
GO and subcellular localization analyses.** A** Prediction of biological process, molecular function and subcellular localization of PpHDZ gene family. Figures represent the percentage of each component. **B** Subcellular localization of three GFP-fused PpHDZ proteins (PpHDZ2-GFP, PpHDZ15-GFP, and PpHDZ16-GFP). The photographs were taken under bright light, in the dark field for the GFP-derived green flourescence and DAPI-derived blue flourescence and merged, respectively

GO ontology annotation was used to analyze and predict the biological processes, molecular functions and subcellular localization of HD-Zip protein in peach (Fig. 5 A). In molecular function, PpHDZ proteins mainly played a role in DNA binding (46.94%) and nucleic acid binding transcription factor activity (46.94%), and a few proteins played a role in protein binding (6.12%). In biological process, PpHDZ members mainly played roles in biosynthetic process (27.38%), cellular nitrogen compound metabolic process (27.38%),anatomical structure development (11.90%) and cell differentiation (9.52%). In addition, PpHDZ members were involved in response to stress (5.95%), reproduction (3.57%), embryo development (3.57%), anatomical structure formation involved in morphogenesis (3.57%), cell morphogenesis (2.38%), signal transduction (2.38%), pigmentation (1.19%), developmental maturation (1.19%) and so on. In terms of subcellular localization, PpHDZ protein was mainly located in the nuclear (78.57%), and a few proteins were located in the cytoplasmic (10.71%) and plasmamembrane (10.71%).

### Subcellular localization of PpHDZ proteins

Nuclear localization of transcription factors is important for regulating transcription of target genes by binding specific cis-elements in their promoters. Previous studies have shown that HD-Zip protein is mainly located in the nucleus. For example, VvHDZ28 in grape is a nuclear protein [21]. In this study, three cloned *PpHDZ* genes (*PpHDZ2*, *PpHDZ15* and *PpHDZ16*) were introduced into the *pCAMBIA1300* vector and fused with *GFP* gene, all under the CaMV35S promoter. The recombinant fusion vector were infiltrated into *Nicotiana benthamiana* leaves. As shown in Fig. 5B (Fig. 5B), the green fluorescence signals of the fusion proteins PpHDZ2-GFP, PpHDZ15-GFP and PpHDZ16-GFP were heterogeneously distributed in the nucleus. This was consistent with the results of GO analysis. The results showed that PpHDZ2, PpHDZ15 and PpHDZ16 were nuclear proteins.

### Promoter element analysis

**Fig. 6:**
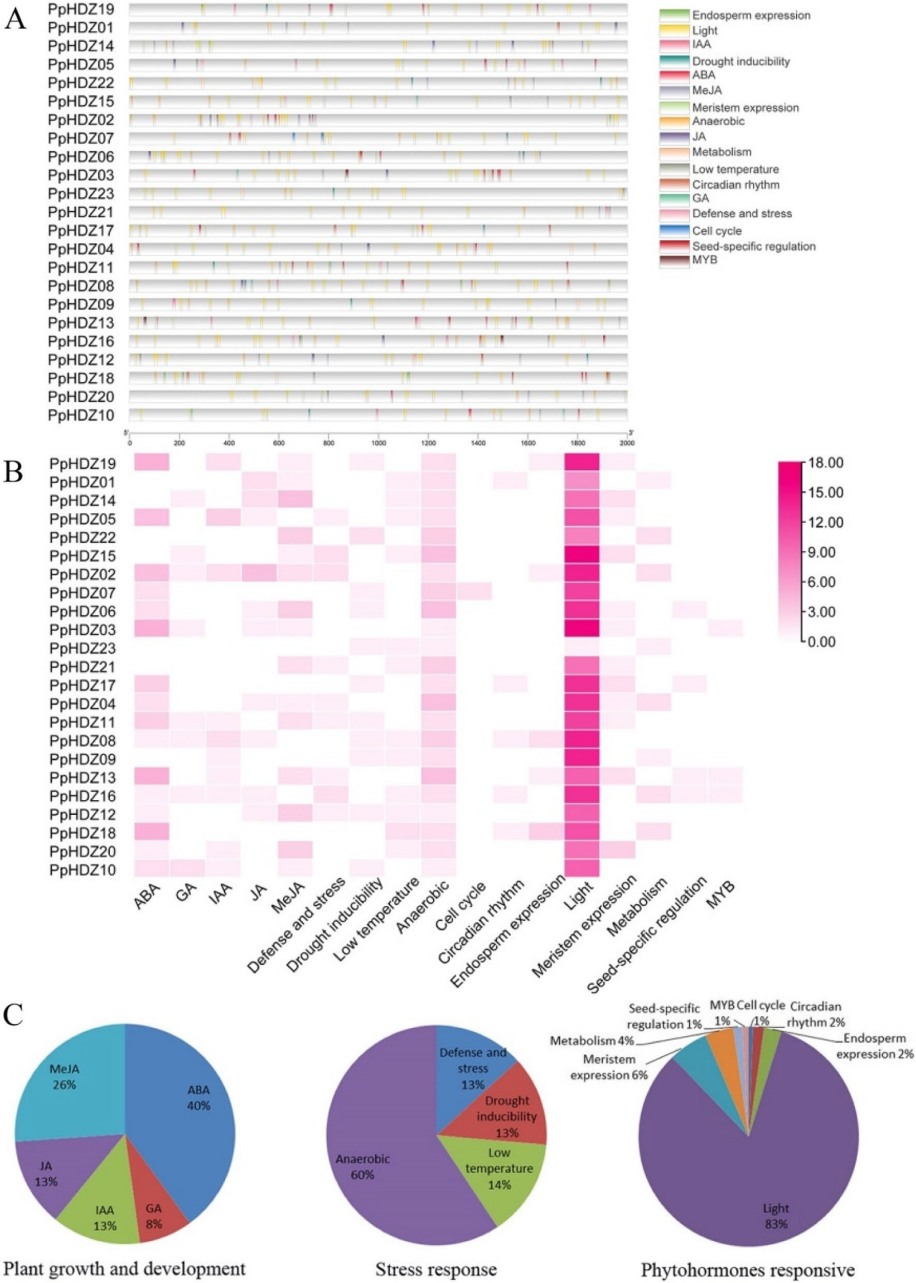
Promoters elements analysis of peach *PpHDZ* genes.** A**. Cis-elements in the location of the *PpHDZ* genes promoter sequences. **B**. Variety of cis-elements of *PpHDZ* gene. The different colors represent the number of each cis-elements. **C**. Distribution of cis-elements of *PpHDZ* gene in plant hormone response, stress response and plant growth and development. Pie charts size represented the percentage of promoter element in each category

The promoter sequences of 23 *PpHDZ* genes (2 kb upstream of 5 ‘UTR) were submitted to PlantCARE database for the prediction of promoter elements and a total of 17 cis-elements were identified (Fig. 6A). Except for two conventional promoter elements TATA-box and CAAT-box, the remaining response elements could be divided into three main categories: plant growth and development, stress response and phytohormones response. The phytohormones response elements in *HD-Zip* gene promoter region mainly included ABA (40%), GA (8%), IAA (13%), JA (13%) and MeJA (26%), indicating that *HD-Zip* genes might be involved in physiological, biochemical and growth and development processes of plants in response to various upstream hormones. Among the stress-related cis-elements, anaerobic induction (60%), drought induction (13%), low temperature induction (14%) and defense stress response (13%) were detected. In plant growth and development related cis-elements, light response elements accounted for the highest proportion (83%), and each peach HD-Zip family member had light response elements, suggesting that the HD-Zip family would play an important role in the establishment of light morphology. Meristem expression response elements and seed development response elements are mainly distributed in the promoters of members of the subfamily III, perhaps because HD-Zip subfamily III is mainly involved in the development of plant embryos and meristem formation [22, 23]. The HD-Zip subfamily IV is mainly involved in the process of substance accumulation [24], so the endosperm expression response elements related to substance accumulation were mainly distributed in the subfamily IV members (Fig. 6B and C).

### Tissue specific expression pattern of *PpHDZ* genes in peach

**Fig. 7:**
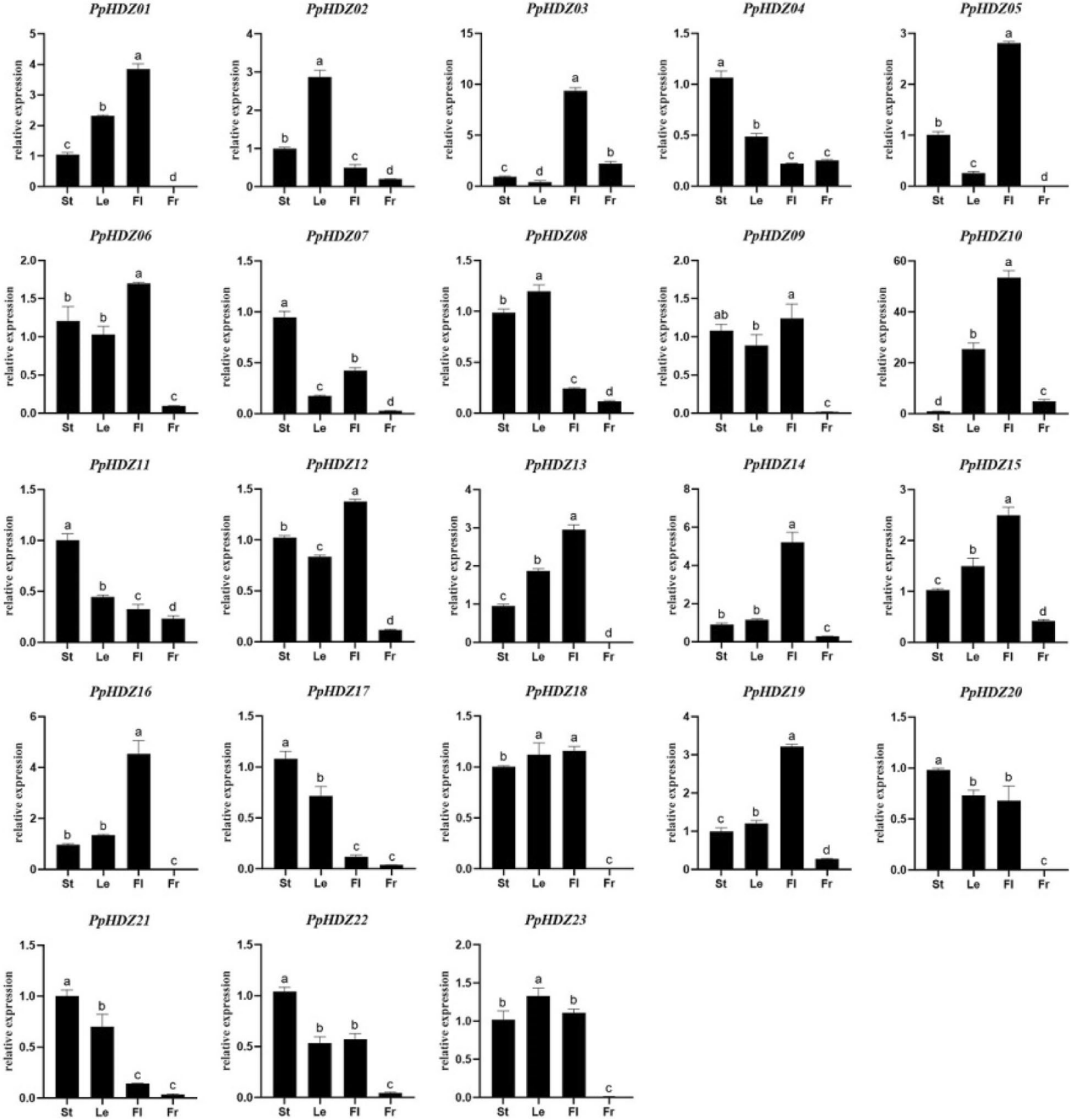
Relative expression of *PpHDZ* genes in different tissues of peach**.**St-Stem, Le-Leaf, Fl-Flower, Fr-Fruit in abscissa. Bars with different letters indicate significant differences (*p*<0.05, Duncan’s multiple range tests). Error bars show the standard error between three biological replicates(*n*=3).

The tissue specific expression pattern of 23 *PpHDZs* was analyzed. Overall, most *PpHDZ* genes exhibited a relatively wide range of expression patterns in a variety of tissues. However, a small number of gene subfamilies were preferentially expressed in specific tissues and developmental processes. The expression levels of subfamilies I and IV were higher in flower organs, indicating that the members of the two subfamilies played an important role in the development of flower organ, which was consistent with previous studies [25, 26]. Subfamily II members were expressed in stem, leaf and flower organ to varying degrees. Subfamily III genes were highly expressed in stem and leaf (Fig. 7), However, the four subfamily members all had low expression levels in peach fruit. The tissue-specific expression of *PpHDZs* suggested that different subfamilies played specific regulatory roles in different tissues.

### Expression pattern of *PpHDZ* genes in different stages of rooting in cuttings

**Fig. 8:**
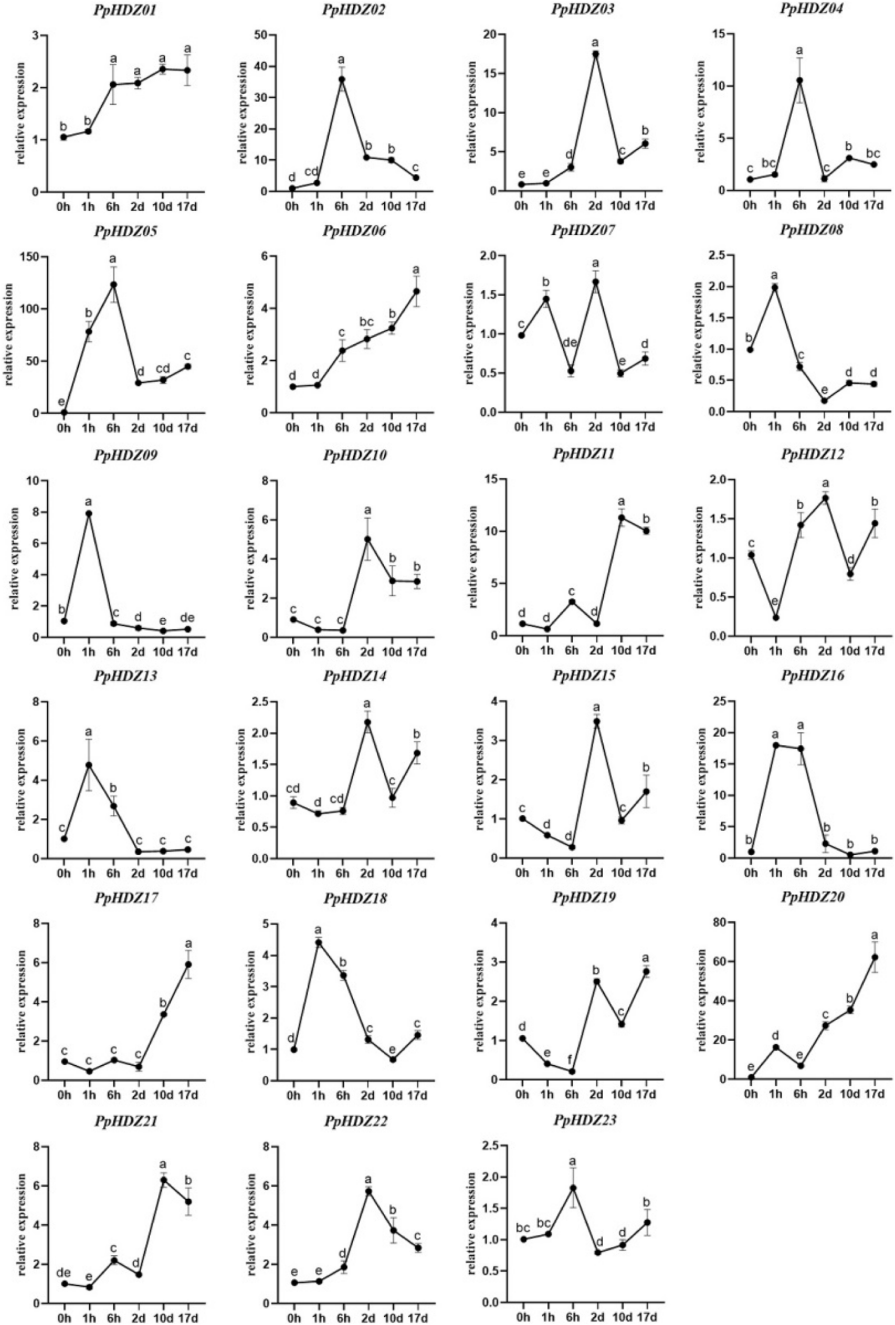
Expression trend of *PpHDZ* genes in adventitious root formation. Different letters indicate significant differences (*p* < 0.05, Duncan’s multiple range tests). Error bars show the standard error between three biological replicates(*n* = 3)

In order to explore the role of *PpHDZ* genes in the formation of adventitious root of cuttings, samples were collected at 0 h, 1 h, 6 h, 2 d, 10 d and 17 d after cuttings respectively, and the expression of the 23 *PpHDZ* genes were determined by qRT-PCR. The results showed that *PpHDZ* genes were expressed differently during adventitious root development. The subfamilies I and II showed high expression mainly in the intermediate stage of adventitious root formation. *PpHDZ08, PpHDZ09, PpHDZ13, PpHDZ16* and *PpHDZ18* of the subfamily IV were strongly expressed in 1 h after cutting, but down-regulated in later cutting period, indicating that they mainly acted in the early stage of adventitious root formation. *PpHDZ06, PpHDZ17*, and *PpHDZ20* were down-regulated in the early stage and up-regulated in the late stage, suggesting that they played a role in the late adventitious root formation (Fig. 8). In addition, the spatio-temporal expression patterns of six homologous genes were analyzed. The expression patterns of *PpHDZ08\PpHDZ09*, *PpHDZ14\PpHDZ15* and *PpHDZ17\PpHDZ21* were similar in different adventitious root formation stages. In contrast, the expression patterns of three pairs of homologous genes *PpHDZ02\PpHDZ06*, *PpHDZ01\PpHDZ19* and *PpHDZ10\PpHDZ20* were significantly different in different adventitious root formation stages (Fig. 8). This suggests that homologous pairs might have undergone differential differentiation during the evolution of *HD-Zip* gene.

## Discussion

Plant specific transcription factor HD-Zip plays an important role in plant growth and development and environmental response. HD-Zip transcription factors have been identified in *Arabidopsis thaliana* [5], *Zea mays* [6], *Oryza sativa* [7], *Populus trichocarpa* [8], and so on. However, there is still no comprehensive and systematic analysis of the HD-Zip gene family of peach, one of the fruit tree crops with high nutritional value in the world. In this study, 23 *PpHDZ* genes were identified from the whole genome of peach. The phylogenetic relationships, gene structure, conserved domains, promoter elements and the expression patterns of 23 *PpHDZ* genes in different tissues and adventitious roots were analyzed. It laid a foundation for further study on the function of *HD-Zip* gene in peach growth and development.

Phylogenetic analysis and multiple sequence alignment revealed that 23 *PpHDZ* genes were mainly divided into four subfamilies, which was consistent with previous results [19]. Moreover, *PpHDZ* genes from the same subfamily have similar conserved domain composition, intron and exon composition, which further supports the evolutionary relationship of 23 *PpHDZ* genes. *HD-Zip* gene is distributed in various plants. And the number of *HD-Zip* gene is similar in *Arabidopsis thaliana* (48 members), *Capsicum annuum* (42 members) and *Carthamus tinctorius* (48 members), which is more than in *Vitis vinifera* (33 members) and *Oryza sativa* (31 members), but much less than in *Zea mays* (55 members) and *Populus trichocarpa* (63 members) [21, 27, 28]. which means that *HD-Zip* genes are amplified to varying degrees in different species. Evolutionary events such as polyploidy and duplication events could increase the gene family members in plants [29, 30]. Collinearity analysis showed that many *HD-Zip* genes in Arabidopsis had corresponding genes in peach, but only 5 pairs of homologous *HD-Zip* genes were also found in peach, According to these results, it concludes that HD-Zip gene family in peach has been more influenced by evolutionary pressures.

More and more studies have shown that the HD-Zip gene family plays an important role in plant growth and development. Such as organ development, environmental response and hormonal response [9]. Based on the annotated information of the Arabidopsis HD-Zip gene family, combined with promoter element analysis and expression analysis of different tissues, the biological function of the peach *PpHDZ* gene could be inferred. For example, *PpHDZ14* was homologous to *AtHDZ06* (*ATHB1*) involved in hypocotyl elongation under short-day condition [31], which indicated that *PpHDZ14* might regulate hypocotyl elongation under short-day condition. Both *AtHDZ19* (*HAT1*) and *AtHDZ27* (*HAT3*) belonging to subfamily II of the evolutionary tree were regulated by changes in light quality and induced by shade avoidance response in plants [32, 33]. There were a high abundance of light response elements in the promoters of Subfamilies I and II, suggesting that the two subfamilies genes of peach might be involved in the formation of light morphology. Moreover, the expression level of most members of subfamily I was higher in peach blossom, indicating that the subfamily I might play roles in peach blossom organs. *AtHDZ29* (*ATHB8*) in subfamily III of the evolutionary tree is involved in vein formation and plant hormone signal transduction [34], suggesting that *PpHDZ04* and *PpHDZ11* belonging to the same subfamily might also have the similar functions. Promoter element analysis revealed that meristem expression response elements were mainly distributed in the genes of subfamily III (Fig. 6B). Expression analysis showed that the genes of subfamily III were highly expressed in stem and leaf (Fig. 7). It could be inferred from these results that subfamily III genes might play a key role in maintaining meristem and be participate in vein formation and hormone transduction. Studies have shown that *HD-Zip* gene may co-regulate the expression of flavonoid compound synthesis genes [35, 36]. MYB elements involved in the regulation of flavonoid biosynthesis genes were found in promoters of *PpHDZ13* and *PpHDZ16* genes in subfamily IV. Expression analysis showed that these two genes were also highly expressed in flowers. *PpHDZ13* and *PpHDZ16* might be involved in the synthesis of flavonoids. *AtHDZ04* (*GL1*), *AtHDZ05* (*HDG11*), and *AtHDZ09* (*HDG12*) are associated with hairy root formation [37, 38], suggesting that subfamily IV might play an important role in trichomes formation. Through evolutionary analysis and tissue expression pattern analysis, the role of the peach HD-Zip gene family could be inferred. Although more experiments would be needed to verify, the study could provide a preliminary theoretical basis for understanding the function of the peach HD-Zip gene family.

The formation of peach adventitious roots is a complex process and a highly coordinated developmental process. Many studies have shown that *HD-Zip* gene is involved in root genesis and development [39, 40]. By detecting the expression levels of 23 *PpHDZ* genes during adventitious root formation, we found that the 23 *PpHDZ* genes were expressed to varying degrees during the adventitious root formation of peach cuttings, and most of the genes were highly expressed in a specific period of peach adventitious root formation (Fig. 8). For example, *PpHDZ08, PpHDZ09, PpHDZ13, PpHDZ16* and *PpHDZ18* were highly expressed in the early stage of adventitious root formation. *PpHDZ02, PpHDZ03, PpHDZ04* and *PpHDZ23* were highly expressed in the medium term. *PpHDZ17* and *PpHDZ20* were highly expressed in late stage (Fig. 8). These results indicated that *HD-Zip* gene played an important role in the morphogenesis and development of adventitious roots of peach cuttings. The expression profile of *PpHDZ* gene is helpful to further understand the functional characteristics of PpHDZ gene family in plant growth and development and adventitious root formation of cuttings.

## Conclusion

In this study, a total of 23 *PpHDZ* genes were identified in peach by genome-wide analysis, distributed on 6 chromosomes. By phylogenetic analysis and conserved domain analysis, *PpHDZ* gene were divided into four subfamilies. A large number of cis-acting elements were found in promoter of *PpHDZ* gene, indicating that *PpHDZ* gene was controlled by a complex regulatory network. The tissue specific expression pattern of *PpHDZ* gene suggested that *PpHDZ* gene might play an important role in peach growth and development. *PpHDZ* gene were expressed variously during the adventitious root formation of peach cuttings, suggesting that PpHDZ would be involved in the formation of peach adventitious roots, and different genes play specific functions in different periods. These results lay a foundation for further exploring the function of *PpHDZ* gene in peach growth and development.

## Materials and methods

### Identification of putative *PpHDZ* in peach

The characteristic domain of HD-Zip transcription factor family protein (PF00046) was obtained from Pfam (Pfam: Home page (xfam.org)). HMMER (Biosequence analysis using profile hidden Markov Models | HMMER (ebi.ac.uk)) was used to construct HMM model to obtain peach HD-Zip transcription factor family candidate protein [41]. NCBI (National Center for Biotechnology Information (nih.gov)) was further used to detect whether the candidate protein contained HD-Zip domain and delete the sequence of the missing domain. Finally, 23 PpHDZ proteins were identified. The molecular weights and isoelectric points of PpHDZ family members were analyzed and identified using the online tool EMBOSS Programs (EMBOSS programs < EMBL-EBI). CELLO2GO (CELLO2GO:Subcellular Localization and Function Analysing System (nctu.edu.tw)) is used to analyze molecular work, biological processes and subcellular localization [42]. And use ChiPlot (bar plot with category (chiplot.online)) to draw GO analysis diagram.

### Chromosomal localization analysis

The annotated files of peach genome (Prunus_persica_NCBIv2) and the length information of peach chromosome were obtained from NCBI (National Center for Biotechnology Information (nih.gov)). The chromosome location information of HD-Zip gene family members was extracted from the annotation file of peach genome, and the peach chromosome location map was drawn by TBtools software, and the HD-Zip family members were labeled on the chromosome [43].

### Phyloevolutionary analysis and collinearity analysis

ClustaLW was used to analyze the amino acid sequences of *Arabidopsis thaliana* and peach HD-Zip [44]. The phylogenetic tree is constructed in MEGA11 by ML (maximum likelihood) method. MEGA11 also calculated the nucleotide differences of *HD-Zip* genes [45]. Genome annotation files of peach (Prunus_persica_NCBIv2) and Arabidopsis (TAIR10.1) were obtained from NCBI (National Center for Biotechnology Information (nih.gov)). Genome-wide collinearity of peach and Arabidopsis was analyzed by MCScanX [46], and genome-wide collinearity was mapped by TBtools.

### Gene structure and conserved domain analysis

The exon and intron location information of *PpHDZ* gene was extracted from peach genome annotation file, and the extracted location information was converted into GSDS2.0 (Gene Structure Display Server 2.0 (gao-lab.org)) readable BED file. The *PpHDZ* gene structure was mapped by GSDS2.0 [47]. MEME5.4.1 (MEME - Submission form (meme-suite.org)) was used to predict and analyze the conserved protein motifs of PpHDZ protein sequences. The searched motif value was set to 15, and other parameters were set as default [48]. The structure diagram of the conserved protein motif was drawn by TBtools.

### Subcellular localization analysis

One *PpHDZ* gene was randomly selected from each of three subfamilies. The nucleotide sequences of the three genes were obtained through the JGI website (Phytozome (doe.gov)) and amplified by P520 polymerase (Vazyme, Dalian, China). Three cloned *PpHDZ* genes coding sequence without stop codon were cloned into *pCAMBIA1300* vector fused with *GFP*, all under CaMV 35 S promoter (35 S:*PpHDZ2-GFP*, 35 S:*PpHDZ15-GFP* and 35 S:*PpHDZ16-GFP*), respectively. These constructs were then transferred into Agrobacterium GV3101, and the transformed Agrobacterium cells were activated and infected into *Nicotiana benthamiana* leaves [49]. After 72 h, GFP was observed using a confocal laser microscope (Zeiss LSM880, Germany) and the nuclei were observed using DAPI-labeled nuclear markers.

### Promoter element analysis

The promoter sequences of each *PpHDZ* gene, 2 kb sequence upstream of 5’UTR, were retrieved from the JGI website (Phytozome (doe.gov)). All the promoter sequences were submitted to the PlantCARE (PlantCARE, a database of plant promoters and their cis-acting regulatory elements (ugent.be)) database for the prediction of promoter elements, and then the distribution map and heat map of promoter elements were drawn using TBtools software [50].

### Plant material acquisition, RNA extraction and qRT-PCR

The peach rootstock ‘GF677’ is planted in the experimental base of Shandong Agricultural University. The tissue samples of ‘GF677’ leaf, stem, flower and fruit were frozen in liquid nitrogen and stored at -80℃. The peach cutting were hardwood shoot of ‘GF677’. The work was carried out in a greenhouse of experimental base of Shandong Agricultural University, on November 12, 2021. The hardwood shoots were collected and cut in to pieces with 4–5 buds, the leaves of base were remove. The upper end of cuttings were cut flat, and the lower end of cutting were cut a 2 cm bevel with a scalpel. The cuttings were put into 1 mg/L IBA solution soaked for 2 min and then inserted into the sand pool. After completion, the sand was watered thoroughly. The sand kept moist by spraying during the whole expreiment. Samples were taken at 0 h, 1 h, 6 h, 2 d, 10 d and 17 d after cutting, and the samples were frozen with liquid nitrogen and stored at − 80 ℃.Total RNA was extracted from the samples using the RNA Plant Plus Reagent Kit (TIANGEN, China) and the first-strand cDNA was synthesized using the PrimeScript First-strand cDNA Synthesis Kit (Takara, Dalian, China). Real-time quantitative polymerase chain reaction (qRT-PCR) was performed in an ABI7500 system using SYBR premix ExTaq (Takara). qRT-PCR was calculated by 2^−ΔΔCT^. At least 3 replicates per sample were used for qPCR [51]. Primers used for qRT-PCR were listed in Table S1.

## Supplementary Information

**Additional file 1: Table S1**. The primers sequences of PpHDZ genes for qRT-PCR and gene cloning. **Table S2**. The Gene IDs of AtHDZ genes. **Figure S1**. The conserved protein motifs in the PpHDZ proteins. The x-axis indicates the conserved sequences of the domain. The height of each letter indicates the conservation of each residue across all proteins. The y-axis is a scale of the relative entropy, which reflects the conservation rateof each amino acid.

## Data Availability

All the data generated or analyzed during this study are included in this published article and its supplementary information files. The peach sequences in this article can be found from phytozome (Phytozome (doe.gov)). The Arabidopsis thaliana sequences in this article were downloaded from TAIR (TAIR - Home Page (arabidopsis.org)). All plant materials were selected from peach provided by the F. Peng lab, Shandong Agricultural University, Taian, China.
