# Supplementary material for: Genome-wide identification, bioinformatics and expression analysis of HD-Zip gene family in peach
Source: BMC Plant Biol. 2023 Mar 2;23:122. doi: 10.1186/s12870-023-04061-w (PMC9979464; doi:10.1186/s12870-023-04061-w)
Supplement: Supplementary file 1 — Additional file 1: Table S1. The primers sequences of PpHDZ genes for qRT-PCR and gene cloning. Table S2. The Gene IDs of AtHDZ genes. Figure S1. The conserved protein motifs in the PpHDZ proteins. The x-axis indicates the conserved sequences of the domain. The height of each letter indicates the conservation of each residue across all proteins. The y-axis is a scale of the relative entropy, which reflects the conservation rateof each amino acid. [file 12870_2023_4061_MOESM1_ESM.docx]

Table S1. The primers sequences of PpHDZ genes for qRT-PCR and gene cloning

| Gene name | Gene ID | Forward primer sequences (5'-3') | Reverse primer sequences (5'-3') | Used |
| --- | --- | --- | --- | --- |
| PpHDZ01 | Prupe.1g267300 | GAACCAGCTGAACAGTCGGA | GGCCAGAAACAAAAGGGCTG | qRT-PCR |
| PpHDZ02 | Prupe.1g325000 | AAAATCGTAGGGCCAGGAGC | GCTCACAACTGTGGGGAGAA | qRT-PCR |
| PpHDZ03 | Prupe.1g416800 | TTGCCTTCTCTTACGCTCGG | AAGAGTCCTGTTCCTGTGCG | qRT-PCR |
| PpHDZ04 | Prupe.1g447100 | TTCTTGTGAACTCGTCCCCG | AGAAGAATGGCTGGAGGCAC | qRT-PCR |
| PpHDZ05 | Prupe.1g523000 | ATTGGGTGTGTTGGTGTCCA | CCTTGACCTGCTCTGAGCTT | qRT-PCR |
| PpHDZ06 | Prupe.2g004000 | AAGCTCCTGCAGAGCCATTT | CTGTCACAGCTTGGACCGAT | qRT-PCR |
| PpHDZ07 | Prupe.2g127300 | GAGAAGCCTTCACAGCCCAT | CCTGGCATCTGGGTTTCGAT | qRT-PCR |
| PpHDZ08 | Prupe.2g156900 | CTCTCACTCGCCCTTCAGAC | ACTCCCAGATCTGCTCTCGT | qRT-PCR |
| PpHDZ09 | Prupe.2g291400 | ATACCCCCTACATGCCCAGT | TTCTCAGCAATCAGGCCCAG | qRT-PCR |
| PpHDZ10 | Prupe.3g015300 | GCGTATGGCGCTGAAAGATG | TGACCATCCTGTGGGAAAGC | qRT-PCR |
| PpHDZ11 | Prupe.3g060700 | CTTAGGTTCCTGCGTGAGCA | CAATAGTGTGAGCCAGGGGG | qRT-PCR |
| PpHDZ12 | Prupe.3g067200 | CAGAAAGCGTCCCTCTGGTT | GGTGCGGTACATGGTCTGAA | qRT-PCR |
| PpHDZ13 | Prupe.3g218500 | AGGCCCTCTGGATGTCTCAT | CCAAGGTAGCAACCCATCGT | qRT-PCR |
| PpHDZ14 | Prupe.3g283200 | AAAGTTGAGGACCGCCTGAG | TGGACGTTGTCCTCGTTGAG | qRT-PCR |
| PpHDZ15 | Prupe.3g269900 | ATGATGGCCTTCCCACCTTG | ACCATGGAAGTGCTGAGGTG | qRT-PCR |
| PpHDZ16 | Prupe.4g024800 | TGCACTTCTGCCATCTGGTT | CTACGGTGAGTAGGGACCCA | qRT-PCR |
| PpHDZ17 | Prupe.4g090100 | CTTGCGGTCTTGTGAGCCTA | CCACCATTTCCAGCAGGGAT | qRT-PCR |
| PpHDZ18 | Prupe.5g029000 | TCGCAGAGCTCAGCTTGATG | GGTGCAGCTCTCTTGTAGCA | qRT-PCR |
| PpHDZ19 | Prupe.5g185900 | TCGCAAAGGACCTAGGCTTG | GTTGGCTTGCAAAACCTCGT | qRT-PCR |
| PpHDZ20 | Prupe.5g240900 | CCGTTGGTACAAACTCGGGA | ATCAAGCACCCGGAAGGAAG | qRT-PCR |
| PpHDZ21 | Prupe.6g102300 | CATGGAGTGTGCCAGAGGTT | CTGTACACCACTTCACCGCT | qRT-PCR |
| PpHDZ22 | Prupe.6g193400 | CCATCTGCCCATCAACAGCAG | CCACGCACCCTTCTTCATCTA | qRT-PCR |
| PpHDZ23 | Prupe.6g213800 | CATAGCGCGGTCTCTTCCTT | GGCAGACTGTTCCTTGGTGA | qRT-PCR |
| PpActin | AB073011 | GTTATTCTTCATCGGCGTCTTCG | CTTCACCATTCCAGTTCCATTGTC | qRT-PCR |
| PpHDZ02 | Prupe.1g325000 | atttggagaggacagggtaccATGGCGGTTTTTCCACCAACA | caccatggtactagtgtcgacGAGGGCGGGCGTTGCCACTTT | Gene Cloning and Vector Construction |
| PpHDZ15 | Prupe.3g269900 | atttggagaggacagggtaccATGATGGCCTTCCCACCTTG | caccatggtactagtgtcgacATGAAAGTGGTGCTGCTCAGG | Gene Cloning and Vector Construction |
| PpHDZ16 | Prupe.4g024800 | atttggagaggacagggtaccATGTATCAGCCAAGCATGTTCG | caccatggtactagtgtcgacTGCGTTCTGTTCACATGTGACC | Gene Cloning and Vector Construction |

Table S2. The Gene IDs of AtHDZ genes

| Gene name | Gene ID |
| --- | --- |
| AtHDZ01 | At4g00730 |
| AtHDZ02 | At5g46880 |
| AtHDZ03 | At1g79840 |
| AtHDZ04 | At3g61150 |
| AtHDZ05 | At1g73360 |
| AtHDZ06 | At3g01470 |
| AtHDZ07 | At4g04890 |
| AtHDZ08 | At4g21750 |
| AtHDZ09 | At1g17920 |
| AtHDZ10 | At5g52170 |
| AtHDZ11 | At1g05230 |
| AtHDZ12 | At4g36740 |
| AtHDZ13 | At2g32370 |
| AtHDZ14 | At1g34650 |
| AtHDZ15 | At2g18550 |
| AtHDZ16 | At4g17710 |
| AtHDZ17 | At5g65310 |
| AtHDZ18 | At4g40060 |
| AtHDZ19 | At4g17460 |
| AtHDZ20 | At3g03260 |
| AtHDZ21 | At2g22430 |
| AtHDZ22 | At5g17320 |
| AtHDZ23 | At5g53980 |
| AtHDZ24 | At1g30490 |
| AtHDZ25 | At5g15150 |
| AtHDZ26 | At1g69780 |
| AtHDZ27 | At3g60390 |
| AtHDZ28 | At1g70920 |
| AtHDZ29 | At4g32880 |


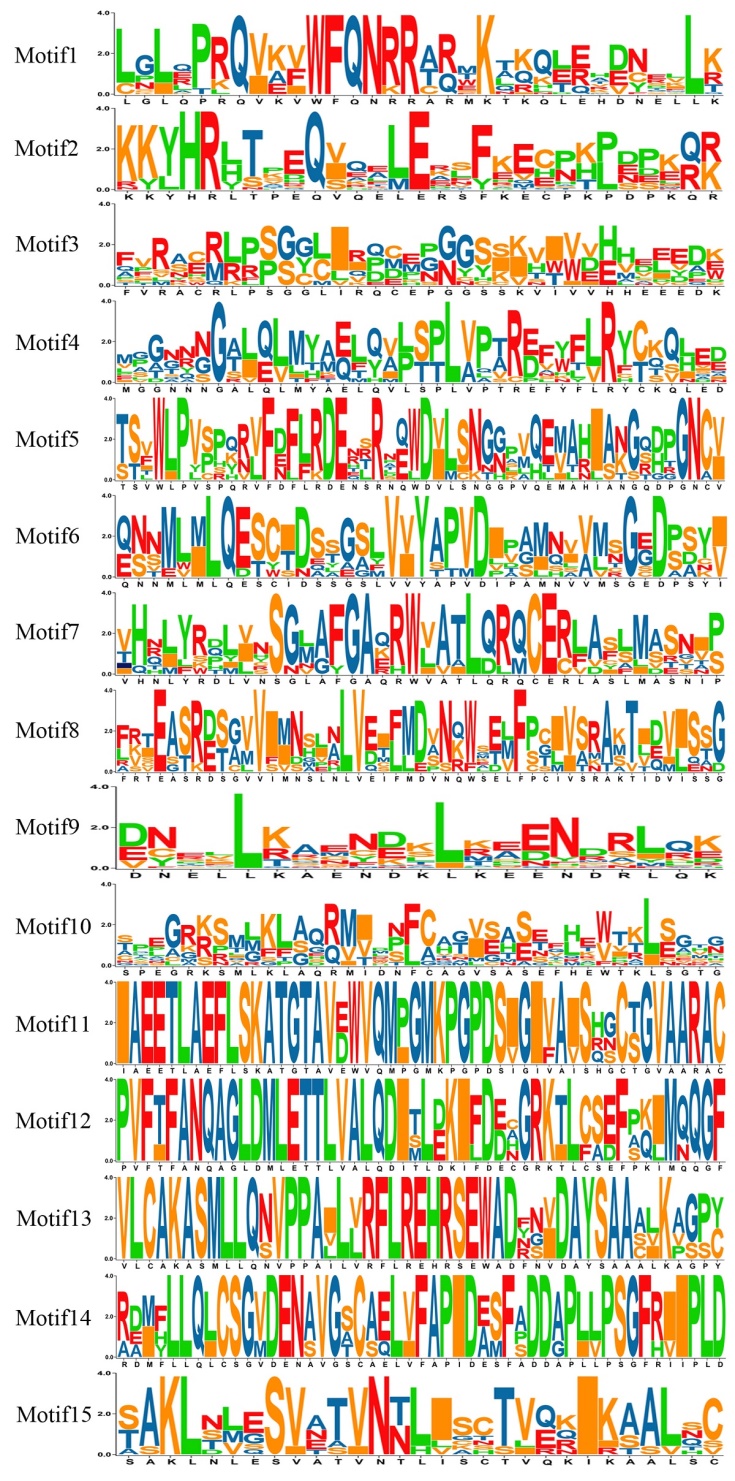


Figure S1. The conserved protein motifs in the PpHDZ proteins. The x-axis indicates the conserved sequences of the domain. The height of each letter indicates the conservation of each residue across all proteins. The y-axis is a scale of the relative entropy, which reflects the conservation rate of each amino acid.
